# Capillary Rise of Nanostructured Microwicks

**DOI:** 10.3390/mi9040153

**Published:** 2018-03-28

**Authors:** Chang-Ho Choi, Shankar Krishnan, Ward TeGrotenhuis, Chih-Hung Chang

**Affiliations:** 1 School of Chemical, Biological, and Environmental Engineering, Oregon State University, Corvallis, OR 97331, USA; choic@engr.orst.edu or aromacch@postech.ac.kr; 2 Department of Chemical Engineering, Pohang University of Science and Technology (POSTECH), Nam-gu, Pohang-Si, Gyungsangbuk-do 37673, Korea; 3 Battelle/Pacific Northwest National Laboratory, MicroProducts Breakthrough Institute, 1000 NE Circle Boulevard, Suite 11101, Corvallis, OR 97330, USA; shankar.krishnan@hotmail.com or kshankar@iitb.ac.in (S.K.); ward.tegrotenhuis@pnnl.gov (W.T.); 4 Department of Mechanical Engineering, Indian Institute of Technology, Bombay, Mumbai 400076, India

**Keywords:** ZnO nanostructure, capillary wicking, ZnO nanoparticle assembly

## Abstract

Capillarity refers to the driving force to propel liquid through small gaps in the absence of external forces, and hence enhanced capillary force has been pursued for various applications. In this study, flower like ZnO nanostructures are successfully deposited to enhance capillarity of microwick structures that are specially designed to augment boiling heat transfer performance. Microreactor-assisted nanomaterial deposition, MAND^TM^, is employed with a flow cell to deposit the ZnO nanostructures on a large sized microwick (4.3 cm × 10.7 cm) with dual-channel configuration. A capillary rise experiment based on the mass gain method is first performed using water and ethanol (EtOH) as the working liquids to demonstrate the enhanced capillary force induced by the ZnO nanostructure on the microwick structure. It is found that the coating of ZnO nanostructure effectively propels the working fluids through the nano- or micro pores created from the ZnO nanostructure and consequently improves the capillary force. In order to investigate the wicking mechanism of the ZnO coated microwick structure, the capillary rise result based on height measurement was compared with analytical models. It is found that the gravity effect and viscous force play an important role in wicking rise of the coated wick structure. This study aims at demonstrating the capability of the integrated MAND process with a flow cell for producing a large scaled nanostructured surface, which eventually has a great potential for enhanced boiling heat transfer.

## 1. Introduction

Capillarity is the ability of liquids to propel through “small gaps” without the assistance of external forces. It has attracted much interest in various fields such as oil recovery, dying of textile fabrics, and ink printing [1,2,3,4]. The phenomenon of capillarity has been investigated in fundamental science and industrial practice as well [5,6,7,8]. Since capillarity concerns the interface that equilibrates the surface energy with gravitational force, it has been used to determine the radius of pores via capillary rise measurements. The Lucas–Washburn equation, formulated from Poiseuille’s law, has been introduced to study the penetration of liquid into porous structures [9]. This approach has been considered a basic instrument for predicting the liquid mobility into porous materials [10]. Recently, rising amounts of criticism regarding the feasibility of the Lucas–Washburn equation have been reported in particular for irregular porous media [11,12,13]. New analytical and numerical models have been developed to account for capillary rise phenomena and are widely applied in models ranging from simple porous structure to complicated structures such as multi-scale porous media [14,15,16]. 

**Figure 1:**
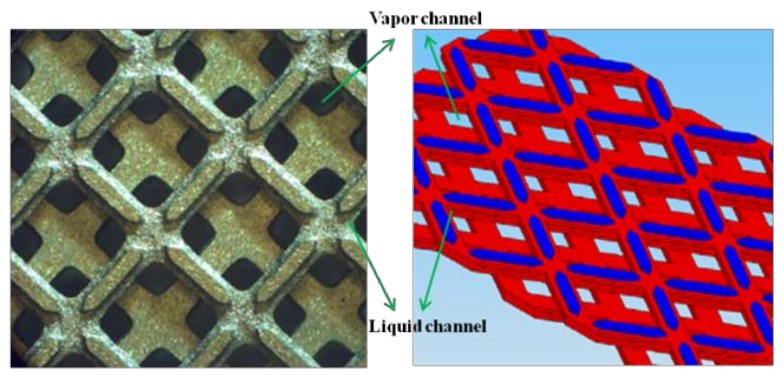
Schematic diagram of dual-mode microwick structures.

It is known that processing gases and liquids together in microchannels having at least one dimension <1 mm has unique advantages for rapid heat and mass transfer. One approach for managing the two phases is to use porous structures as wicks within microchannels to segregate the liquid phase from the gas phase. At the Pacific Northwest National Laboratory (PNNL), gas-liquid processing is accomplished by providing a gas flow path and a separate flow for the liquid phase through the wick under an induced pressure gradient [17]. These microwick technologies enable a variety of applications including phase change, heat exchange, phase separation, partial condensation, absorption, desorption, and distillation [18,19,20]. New wicking structures are being developed at PNNL that are capable of wicking liquid and accommodating vapor flow in the same space [21]. These structures are referred to as ‘dual-mode’ wicks because they display multiple time scales and length scales in wicking fluids (Figure 1). These porous structures enabling wicking evaporators displayed boiling heat transfer enhancement [22]. The interwoven liquid and vapor paths in dual-mode wicks facilitated phase segregation and suppressed or delayed dry out of heated surfaces. Experimental results showed heat transfer coefficients exceeding 20,000 W/m^2^ K. Other characteristics demonstrated in [22] include reduced pressure fluctuations, and lower superheat requirements when compared to plain channels.

Wettability of these microwick structures plays an important role in terms of optimizing the performance of the microwick structure, particularly for the boiling heat transfer. In our prior work, ×3 enhancement in critical heat flux (CHF) and ×10 in boiling heat transfer coefficient were observed in pool boiling experiments with hydrophilic surfaces [23]. We have developed unique hydrophilic surfaces by coating ZnO nanostructures having dual-scale surface roughness on various plain substrates. Contact angles of less than 20° have been observed on aluminum substrates coated with nanostructures (versus 104° on bare aluminum substrates).

In this study, the microwick structures (4.3 cm × 10.7 cm) are coated with flower like ZnO nanostructures by using a microreactor-assisted nanomaterial deposition (MAND^TM^) technique with a flow cell to enhance the capillarity of the microwick structure. The liquid mass gain method is conducted to demonstrate the enhancement of wicking rise in the ZnO coated microwick structure using water and ethanol as the working liquids. Some analytical models are presented and compared with the experimental capillary rise to determine the model that best accounts for the enhanced capillary rise of EtOH in the coated microwick structure.

## 2. Materials and Methods

### 2.1. Deposition of Flower Like ZnO Nanostructure on the Microwick Structure

Flower like ZnO nanostructures were deposited on the microwick structure by using the MAND technique and a flow cell. The microwick structure that consists of liquid and vapor channel is shown in Figure 1. The dimension of the microwick structure is also given in Table 1. ZnO is a versatile material with many potential applications including light emitting diodes, field effect transistors, ultraviolet lasers, chemical sensors, and solar cells [24,25,26,27]. Since size and morphology of ZnO nanostructures play an important role in determining physical properties, numerous efforts have been made to control the size and morphology. In a solution based synthetic technique, solution conditions such as pH, temperature, and concentration of precursor lead to various ZnO morphologies including rod-like, prism, and flower like structures [28,29,30]. These structures were generally obtained by the hydrothermal and solvothermal technique with at least several hours reaction time. The modification of the structures is a result of the dissolution–crystallization mechanism during the aging process. In the MAND technique, on the other hand, the growth mechanism is very distinct from the conventional growth mechanism. The MAND technique has several unique effects over the conventional growth approaches on the preparation of nanocrystals and nanostructured surfaces. (1) The microreactor helps the homogenous nucleation and growth of the nanocrystals by drawing homogeneous mixing of reactant and minimizing pH and temperature gradient in the solution. (2) The MAND technique enables the nanocrystal growth process to be tailored by controlling system parameters such as reaction temperature and fluid flow of the solution, leading to faster and more efficient preparation of nanocrystals. (3) Lastly, we can fabricate various nanostructured surfaces via direct delivery of nanocrystals on targeted substrates.

**Figure 2:**
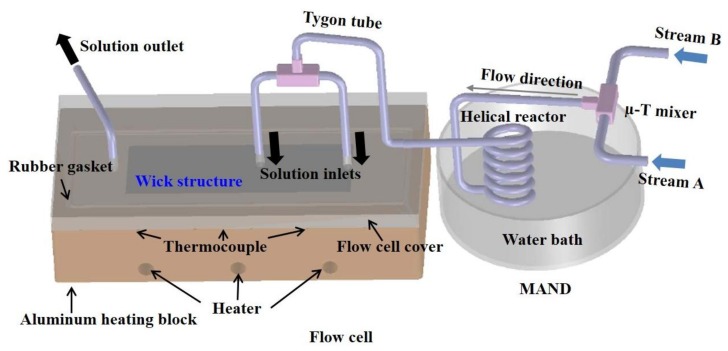
Schematic diagram of integrated microreactor-assisted nanomaterial deposition (MAND) process with a flow cell.

The schematic diagram of the MAND system with the flow cell is shown in Figure 2. The MAND system mainly consists of a micro T-mixer (Upchurch Scientific Inc., Oak Harbor, WA, USA) to enhance mixing of the reactants, a helical reactor made by wrapping a 5 ft long Tygon ST tubing (1.2 mm ID, Upchurch Scientific) around a cylinder, and a water bath to adjust reaction temperature. A flow cell made of an aluminum block was specifically designed to accommodate the microwick structure for the deposition. Three cartridge heaters were embedded in the flow cell to provide uniform heat distribution, and heat flux was controlled by a variac. Reaction temperature during the deposition was monitored by three thermocouples placed nearby the deposition area. The temperature variation was interpreted via LabVIEW software (LabVIEW 8.5, National Instruments (NI), Austin, TX, USA). The cover was made of a transparent polycarbonate material that allows for the observation of the deposition process. The flow cell was tightly sealed with a rubber gasket and clamps to prevent any possible leaking of solution. Three holes on the cover provided two inlet streams and one outlet stream for the solution flux. 

The reactants, zinc acetate (Zn(CH_3_COO)_2_·2H_2_O, Sigma Aldrich, St. Louis, MO, USA), ammonium acetate (CH_3_COONH_4_, Mallinckrodt Chemicals, St. Louis, MO, USA), and sodium hydroxide (NaOH, Mallinckrodt Chemicals, St. Louis, MO, USA), were used as received without further purification. For the ZnO nanocrystal synthesis and the deposition of ZnO nanostructures on the microwick shim, stream A composed of 0.005 M zinc acetate and 0.25 M ammonium acetate solution and stream B (0.05 M NaOH) were initially pumped into the Tygon tubing and allowed to mix homogeneously in a micro-T-mixer. The solution mixture of A and B then passed through a 5 ft long helical structured reactor. By immersing the reactor in the water bath, the reaction temperature (70 °C) was maintained throughout the growth process. The solution exiting from the helical reactor entered another T-mixer where one stream solution was divided into two streams to equally feed reacting solution onto the large scaled microwick structure. The solution then passed through the flow cell maintained at constant temperature (70 °C). ZnO nanostructures were deposited onto the microwick structures as the solution flowed over the microwick structure. The solution flowed out through the outlet port. Deionized water (DI) water was used throughout the experiment and vigorously degassed to remove dissolved air prior to the deposition. The vigorous degassing process is necessary to prevent bubble creation during the deposition, which would increase the pressure drop inside the flow cell and cause irregular deposition. Flow rate of the solution adopted for the nanostructure deposition was 14.7 mL/min.

### 2.2. Capillary Rise Measurement—Height Measurement Approach and Mass Gain Approach

The capillary rise measurement was performed at room temperature. The coated wick structure was vigorously cleaned with acetone, ethanol and DI water in an ultrasonic bath to remove contaminant prior to the wicking measurement. Then, the wick structure was completely dried with nitrogen gas. 

Capillary rise was characterized by height variation with respect to time. This capillary rise measurement involved placing the wick structure on end into the liquid that is wetting for the material. The wick structure was lowered until the end of the structure was wet by the liquid. At time *t* = 0, the wicking sample made contact with the liquid. The liquid front was visually seen with the naked eye. The liquid penetration into the structures was recorded by high resolution HD camcorder (Canon HG10, 6.1megafixel resolution, Melville, NY, USA) until it reached steady state. Recording was performed with the rate of 30 frames per second, and the liquid front was analyzed with a video editing software, Final Cut Pro (Final Cut Pro 7.0.3, Macromedia Inc., San Francisco, SF, USA). Because the wicking front was not even due to the irregular pore size of the coated wick structure, average height of wicking front over the width of the structure was estimated and used to extract the plot of the experimental capillary rise. 

Capillary rise was also characterized by measuring the mass variation of the microwick structures as a function of time. The wick structure was directly connected to a programmable scale (OHAUS, EP114C, Parsippany, NJ, USA). Working liquid was slowly lifted up by using a height controller until the liquid wetted the wick structure. The mass variation was monitored by the fine scale and saved by LABVIEW software every 0.3 s. Like the height measurement method, time *t* = 0 was taken as the microwick structures were brought into the contact with the liquid. Weight of working liquid was recorded from the initial wetting of the wick structure to the complete evaporation of working liquid after detachment of the wick structure from the liquid. 

## 3. Results and Discussion

### 3.1. Deposition of the Flower Like ZnO Nanostructure on the Microwick Structure

**Figure 3:**
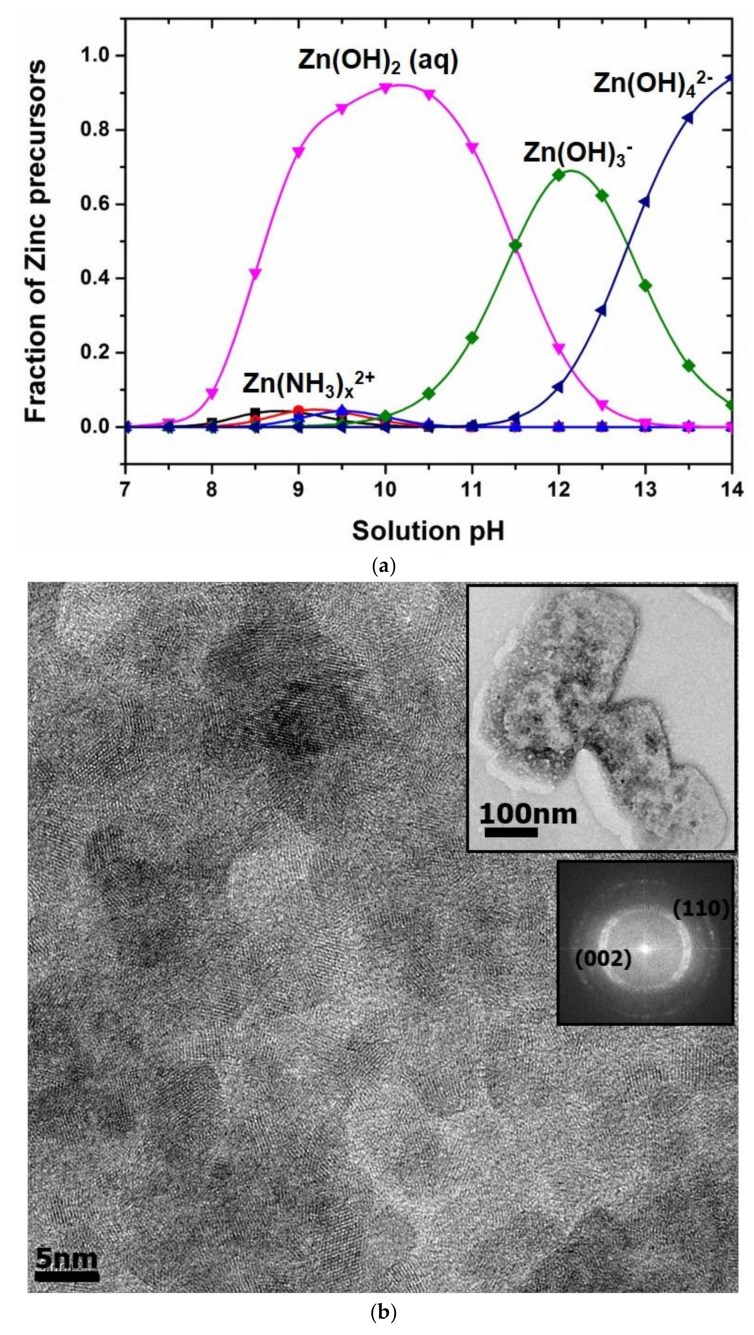
(**a**) speciation diagram of Zinc precursors; (**b**) TEM image of ZnO nanocrystals assembled to form rectangular structure in the MAND system (inset: TEM image of rectangular ZnO assembly and FFT image of assembled ZnO nanocrystals).

**Figure 4:**
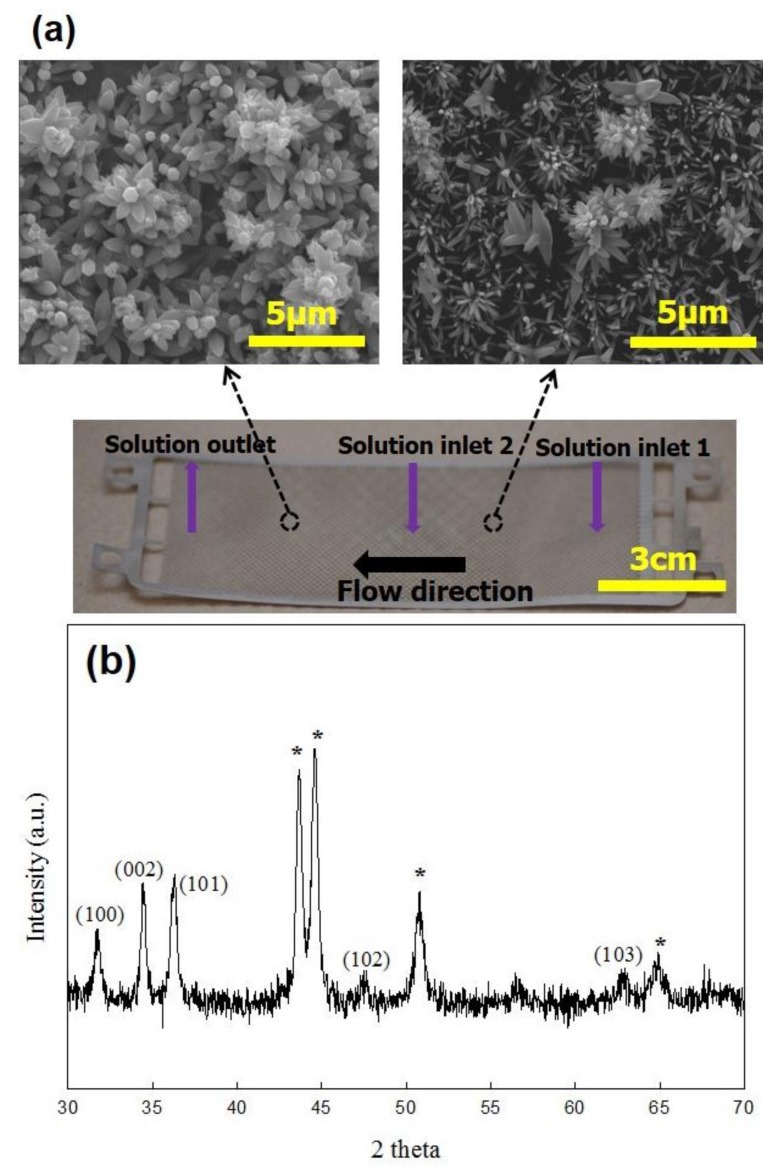
(**a**) SEM images of ZnO nanostructures grown on the wick structure and photograph of the coated wick structure; (**b**) X-ray diffraction pattern of the coated wick structure (* denotes stainless steel peaks from bare wick structure).

The speciation diagram of zinc precursors was constructed as a function of solution pH by using Visual MINTEQ software (Visual MINTEQ 3.1, KTH, Stockholm, Sweden) as shown in Figure 3a. Solution acidity for the growth of ZnO nanocrystals was measured to be around pH = 12.5 where Zn(OH)_3_^−^ is a dominant precursor. Primary ZnO nanocrystals were formed by the condensation reaction of the precursors. The primary ZnO nanocrystals then randomly aggregated to form the rectangular ZnO assembly with around 200 nm in width and 400 nm in length (Figure 3b). A Fast Fourier transform (FFT) image of the ZnO assembly shows polycrystalline ZnO, indicating that the assembly was formed by aggregation of colloidal ZnO nanocrystals. The clear lattice fringes of colloidal ZnO nanocrystals in Figure 3b also confirms the rectangular ZnO assembly formed by the aggregation of individual ZnO nanocrystals. It is hypothesized that the rectangular ZnO assembly synthesized in the MAND process was deposited onto the microwick structure secured in the flow cell and served as a seed layer for the subsequent growth of flower like ZnO nanostructure. It was reported in our previous study that colloidal ZnO nanocrystals synthesized in the MAND process could aggregate together to from assembled ZnO structure with a certain shape, and its shape was dependent upon the flow rate [31]. We also demonstrated the in situ fabrication of ZnO nanostructured surfaces on a SiO_2_ substrate with 4 cm^2^ area by directly delivering the assembled ZnO structure onto the substrate in the MAND technique [32]. Different from the previous study, a flow cell should be integrated with the MAND process for coating of ZnO nanostructure on the large scaled microwick structure (4.3 cm × 10.7 cm) with dual-channel configuration. A flow cell affords the ZnO deposition on the large scaled microwick structure by accommodating the microwick structure and enabling the growth of ZnO nanostructure under continuous feeding of the reacting solution over the microwick structure. Figure 4 shows SEM images of flower like ZnO nanostructure in different regions and the photograph of the coated wick structure. Size and shape of ZnO nanostructures were varied upon the position of the wick structure. This may be due to the fact that more ZnO nanostructures near the solution outlet have the assembled ZnO structure for the growth of flower like structure than those near the solution inlet. For the growth of the nanostructure near the solution outlet area, ZnO assembly from solution inlet 2 and the remnant of ZnO assembly from solution inlet 1 may participate into forming ZnO flower like structure. It would be very challenging to achieve uniform nanostructure coating on the large sized microwick structure (4.3 cm × 10.7 cm) with the dual-channel configuration. Although the size of the ZnO nanostructure was broad, the nano- or micro pores were formed, satisfactorily providing the channel for working liquid to penetrate upward with enhanced capillary force. Porosity (ϕ) of the ZnO nanostructured microwick is a key parameter to evaluate and predict the capillary force and thus needs to be estimated. The traditional method such as gas adsorption is unsuitable for the porosity measurement of the ZnO nanostructures coated on the microwick due to the very small amount of material. The sufficient amount material for the gas adsorption could be obtained by synthesizing powdery flower like ZnO nanostructure in the MAND process. However, the morphology of ZnO nanostructures synthesized homogeneously in the MAND process would differ from that heterogeneously formed on the microwick, giving inaccurate porosity information. Therefore, we estimated the porosity of the ZnO nanostructure coated on the microwick by estimating the ratio of actual density of the coated wick structure and bulk density measured from dimension of the bare wick structure with free of pores [33]. The resulting porosity of the ZnO nanostructures on the microwick was estimated to be 58% and used for wicking mechanism study. The X-ray diffraction pattern of the coated wick structure is well matched with reported one, confirming that the rectangular ZnO assembly served as a building block for the formation of the flower like ZnO structure (Figure 4b) [32].

### 3.2. Capillary Rise Experiments of the Coated Wick Structure

**Figure 5:**
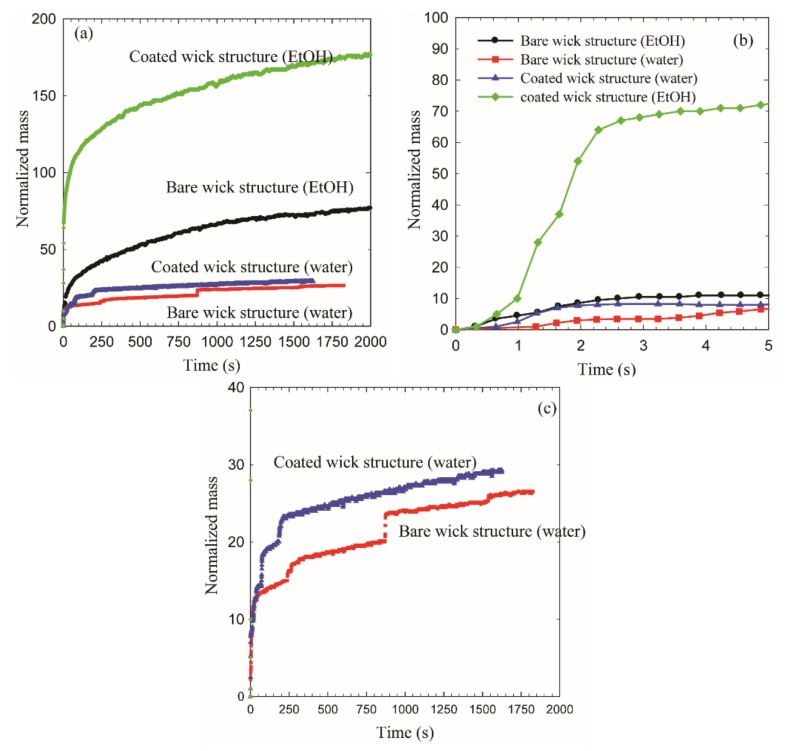
Capillary rise by mass gain approach; (**a**) comparison of all tested wick structure; (**b**) capillary rate at initial wicking; (**c**) closer look of step-rise wicking rise with water as a working fluid.

**Figure 6:**
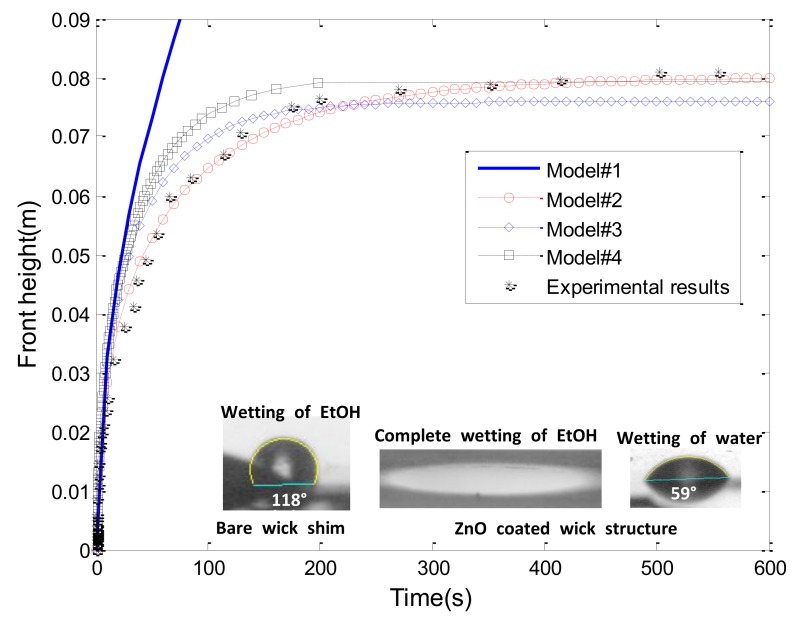
Comparison of experimental wicking results of ZnO coated wick structure with analytical models (inset: static contact angle measurement of EtOH and water on bare wick shim and ZnO coated wick structure).

Figure 5 exhibits the capillary rise obtained by the mass gain approach. Both bare wick structure and ZnO coated wick structure were tested with the use of water and EtOH to demonstrate the enhanced capillary rise of the ZnO coated wick structure. It should be noted that mass uptake plotted on the vertical axis is normalized with dry weight of the wick. It was observed that the capillary rise of the coated wick structure is enhanced for both working liquids, as compared to that of the bare wick structure. The nanostructure coating effects on the capillary rise are more pronounced when EtOH is used as the working liquid compared to water. Thus, the most efficient capillary rise can be obtained using the coated wick structure with EtOH. The capillary rise phenomenon is governed by multidisciplinary factors including physical properties of the working fluid, structure of the wicking substance, and interaction between working fluid and wicking substance. In typical, wettability of the working fluid on the surface plays an important role in determining the capillary rise according to the formulation of the capillary force. The static contact angle of EtOH on the coated microwick structure is lower than that of water, which could be a primary factor to cause the capillarity difference between EtOH and water (inset in Figure 6). The capillary rise at initial contact regime is shown in Figure 5b. Wicking rate of the coated wick structure in EtOH is about ×7 larger than that of the bare wick structure. Wicking rate is an important characteristic in filling the liquid into porous media particularly for absorbing technology [34]. The enhanced wicking performance of the coated wick structure clearly indicates that the nano- or micro-pores as well as improved wettability generated by the flower like ZnO nanostructures propels the working fluid more effectively as compared to the bare wick structure. More details on capillary rise of the ZnO coated microwick structure will be discussed later in the mechanism study.

A unique characteristic of the capillary rise with water is displayed in Figure 5c. The capillary front of water penetrated into the liquid channel of the microwick structure showing step-rise increment. This may be due to the presence of periodically varying pores as discussed in the literature [35].

We confirmed the enhanced capillary rise of EtOH in the ZnO coated wick structure. In order to investigate the mechanism of its wicking rise, we carried out the height measurement capillarity of EtOH in the ZnO coated wick structure and compared the resulting measurement with analytical models. Figure 6 shows temporal evolution of the capillary rise height of EtOH for nanocoated microwick structures. The capillary rise height reached a steady state of about ~500 s. The equilibrium height at the steady state was 80 mm. In order to investigate the parameters that affect the capillary rise of the coated wick structure, the height measurement result is compared with the analytical models as shown in Figure 6. Several analytical models have been proposed to predict the capillary rise and widely used to investigate the wicking process of regular or irregular porous systems. 

(1) The Lucas–Washburn (L–W) equation is the simplest formation by taking negligible gravitational and evaporation effects [9]. Capillary force is solely balanced with viscous force:

(1) $$\begin{array}{c} \frac{2\sigma \cos{ϴ}_{s}}{R_{s}}=\frac{\emptyset }{K}µh\dot{h},\\ h^{2}=\frac{4\sigma \cos ϴ}{\emptyset µ}\frac{K}{R_{s}}t. \end{array}$$

Equation (1) is the L–W equation expressed with permeability (*K*) and porosity by correlating with the Darcy’s law.

(2) Fries et al. recently reported a model accounting for the gravitational effect and viscous force with negligible evaporation effects [36]. Capillary force is balanced with gravity and viscous force:

(2) $$\begin{array}{c} \frac{2\sigma \cos{ϴ}_{s}}{R_{s}}=\rho gh+\frac{\emptyset }{K}µh\dot{h,}\\ h\left(t\right)=-\frac{a}{b}\left[1+W\left(-e^{-1-\frac{b^{2}t\;}{a}}\right)\right],\\ a=\frac{2{\sigma \cos\theta }_{s}}{R_{s}},\;b=-\frac{\rho Kg}{\emptyset µ}. \end{array}$$

The Lambert *W* function was used to extract the wicking rise as a function of wicking time.

(3) The model accounting for evaporation effect and viscous force was also proposed by Rogacs et al. [37]. Capillary rise level out viscous force and evaporation effect:

(3) $$\begin{array}{c} \frac{2{\sigma \cos\theta }_{s}}{R_{s}}=\frac{\emptyset }{K}\mu h\dot{h}+\frac{\mu {\dot{m}}_{e}}{2d\rho K}h^{2},\\ h\left(t\right)=\sqrt{\frac{a}{c}\left(e^{2ct}-1\right),}\\ c=\frac{-{\dot{m}}_{e}}{2d\rho \emptyset }. \end{array}$$

(4) The full and implicit model was reported by Fries et al. to depict all effects such as capillary rise, gravity force, viscous pressure loss, and evaporation effects [38]:

(4) $$\begin{array}{c} \frac{2\sigma {\cos\theta }_{s}}{R_{s}}=\rho gh+\frac{\emptyset }{K}\mu h\dot{h}+\frac{\mu {\dot{m}}_{e}}{2d\rho K}h^{2},\\ t=\frac{1}{2c}\left(\ln\left|\frac{ch^{2}+bh+a}{a}\right|\right)-\frac{b}{2c\sqrt{b^{2}-4ac}}\left(\ln\left|\frac{\left(2ch+b-\sqrt{b^{2}-4ac}\right)\left(b-\sqrt{b^{2}-4ac}\right)}{\left(2ch+b+\sqrt{b^{2}-4ac}\right)\left(b+\sqrt{b^{2}-4ac}\right)}\right|\right). \end{array}$$

In Figure 6, the model 1 (the L–W equation) is much deviated from the experimental capillary rise. Such a deviation is anticipated due to the fact that gravity effect has an influence on the capillary rise of the wick structure. It is generally known that the gravity effect is only negligible at the initial rise of working liquid. Nevertheless, the L–W equation is very useful to determine the pore parameters (*K*/*R_s_*) of the coated wick structure by incorporation of the experimental capillary rise. Because evaporation and gravity effect can be negligible at the initial capillary rise, the pore parameter can be extracted by analyzing the initial capillary rise regime with known physical variables in the L–W equation (model 1). In addition, it was reported that the L–W equation is valid for the wicking rise up to 10% of equilibrium wicking height [36]. The value of the pore parameter (*K*/*R_s_*) can be applied to plot all of the analytical models. Porosity (ϕ) of 58 % was used, and contact angle was considered 0° based on the static contact angle measurement made on the plane area of the coated wick structure (cos θ = 1) (inset in Figure 6).

Model 2 accounts for the capillary rise with gravity effect and viscous force with the negligible evaporation effect. The Lambert W function was solved with given and calculated variables by using MATLAB software (MATKAB 2010b, MathWorks, Natick, MA, USA). The plot of model 2 revealed the excellent agreement with the experimental capillary rise. 

**Figure 7:**
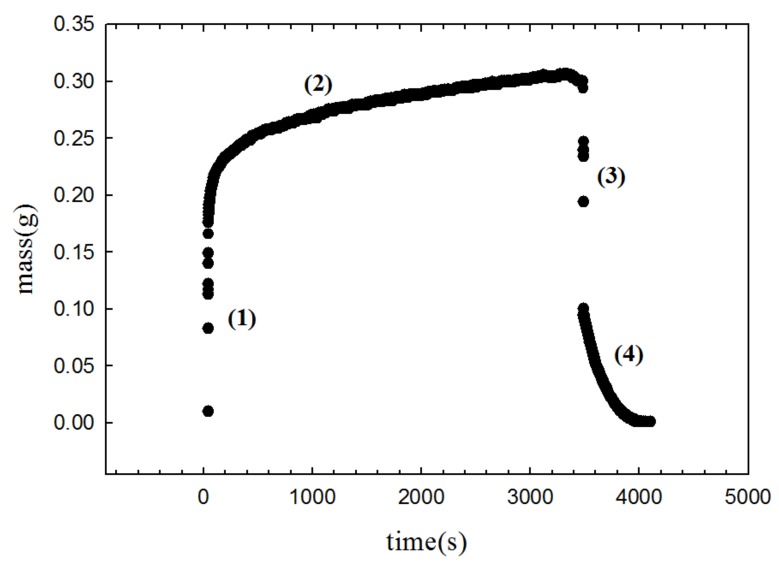
Mass gain approach of the coated wick structure in EtOH; (**1**) wetting regime; (**2**) wicking regime; (**3**) de-wetting regime; and (**4**) evaporation regime.

Rogacs et al. proposed the analytical model that explains evaporation effect with negligible gravity influence (model 3). Model 3 requires the evaporation rate (*m_e_*), which can be estimated by analyzing results of the mass gain approach. Figure 7 shows the wetting and de-wetting dynamics of EtOH in the coated wick structure measured by the mass gain approach. Four distinct regimes are evident in Figure 7. As the wick structure was brought into the liquid, the liquid wetted the wick structure (1). Initially, due to capillary pressure, the wicking rise accelerated (gravity has negligible influence in this regime). Liquid-uptake continued to increase with time and eventually attained a steady state when the capillary pressure, viscous force, and evaporation effect reached an equilibrium state (2). Once the mass uptake reached a steady state, the wick structure was lifted up, referred to as the de-wetting regime (3). Then, the wetted wick structure was left in an open chamber (i.e., unsaturated environment) until the evaporation process was completed (mass = 0) (4). The evaporation regime provides an important key to determine the mass flow due to the evaporation effects. The mass of evaporated liquid per area and time (${\dot{m}}_{e})$ was calculated by analyzing the evaporation regime, and this mass value was used to obtain the mass flow rate due to evaporation $\left({\dot{M}}_{e}\right)$ [38]. The calculated mass flow rate due to the evaporation was about 2.62 × 10^−7^ kg/s while the mass flow rate for the capillary rise was about 1.60 × 10^−6^ kg/s, which means that evaporation affects the capillary rise of EtOH in the nanocoated wick structure (about 16.3% of mass is evaporated). With the estimated evaporation rate (${\dot{m}}_{e}),$ the analytical model 3 was plotted in Figure 6. It was revealed that model 3 is deviated from the experimental results especially when the liquid becomes the equilibrium state. This confirms the role of the gravity effects in the wicking rise of the coated wick structure. Rogacs et al. have developed the model 3 to characterize the wettability of nanostructured porous thin film (~10 µm film thickness) made by hydrophilic silicon nanowire arrays [37]. They reported a simple criterion to determine whether evaporation or gravity effects can be ignored, which depended on sample sizes and experimental conditions. The valid film dimension for the negligible gravity effects was reported to be less than 10 µm film thickness and less than 4 cm wicking height. In our wick structure, the film thickness is less than 10 µm because the typical height of the flower petal was around 1 µm. However, as shown in Figure 6, the maximum height was around 8 cm, which is about ×2 larger than a typical thin film suitable for negligible gravity effects. Therefore, in our wicking rise of the coated wick structure, the gravity effects should be considered. A comprehensive model 4 is also compared with the experimental results in Figure 6. Model 4 is somewhat deviated from the experimental capillary rise. Fries et al. reported the average deviation between model 4 and their experimental results to be around 20%. The factors causing such deviation were also demonstrated [38]. The ambiguity of model 4 was also found in the extraction of certain wicking parameters under typical experimental conditions [37].

Based on the comparison of the experimental wicking rise with the analytical models, we can determine the relative importance of evaporation and gravity effects on the wicking rise of the coated wick structure. The gravity force was more influential than evaporation effect for our wicking measurement although evaporation was practically observed, which leads to better agreement of model 2 than model 3. The minor discrepancy between model 2 and experimental data may be caused by the inaccuracy of height analysis and evaporation effect as well. The variables used to solve the analytical models are presented in Table 2.

The ZnO nanostructured microwick shim was demonstrated to improve the capillary rise as compared to the bare wick shim, particularly as EtOH was used as a working fluid. The flower-like ZnO nanostructures deposited on the wick shim improved wettability and created nano- or micro pores, resulting in the promotion of wicking rise. According to the mechanism study of the capillary rise, gravity and viscous force were most influential forces to balance the capillary force. This study demonstrates that the combination of the MAND process with a flow cell is capable of depositing ZnO nanostructure on a large surface area, especially for wick shim structure, offering a potential application for enhanced boiling heat transfer.

## 4. Conclusions

Flower like ZnO nanostructures were deposited onto the microwick structure by integrating the MAND technique with the flow cell. Assembled ZnO nanocrystals synthesized in the MAND process were used as the building blocks for the growth of ZnO nanostructure on the microwick structure. The mass gain approach was performed to demonstrate the role of the ZnO nanostructure in enhancing the capillary force. The improved wettability of working fluids and the creation of nano- or micro pores were attributed to the flower like ZnO structure, resulting in the enhancement of capillary rise. The results of the EtOH wicking rise in the ZnO coated microwick structure were compared with analytical models to investigate the mechanism of the capillary rise in the coated microwick structure. It was found that gravity effect and viscous force are the most influential forces that balance with the capillary force of the coated wick structure.

## Figures and Tables

**Table 1:** Geometrical factors of the microwick structure.

Width (cm)	Length (cm)	Thickness (µm)	Hydraulic Diameter of Liquid Channel (µm)	Ratio of Vapor to Liquid Volume
3.81	10.2	102	63	2.5~3

**Table 2:** Physical properties of EtOH used for capillary rise study at 19 °C.

Symbol	Value
σ*_lv_* [N/m]	0.0239
cos ϴ	1
ρ [kg/m^3^]	789
*g* [m/s^2^]	9.8
µ [NS/m^2^]	0.0012

## Nomenclature

σ*_lv_* [N/m]	Surface tension	*R* [µm]	Pore radius
cos ϴ	static contact angle	*K* [m^2^]	Permeability
ρ [kg/m^3^]	density	ϕ	Porosity
*g* [m/s^2^]	gravity	*m_e_* [kg/m^2^s]	Mass flow rate by evaporation
µ [NS/m^2^]	dynamic viscosity	*d* [m]	film thickness
*h* [m]	location of meniscus	*t* [s]	time
